# The complete chloroplast genome sequence of *Trikeraia hookeri*

**DOI:** 10.1080/23802359.2021.1961621

**Published:** 2021-08-18

**Authors:** Demei Liu, Liu Ruijuan, Shihai Yang, Xiang Li, Yemeng Zhang, Lili Zhu, Yushou Ma

**Affiliations:** a China and Qinghai Provincial Key Laboratory of Crop Molecular Breeding Northwest Institute of Plateau Biology, Chinese Academy of Sciences, Xining, Qinghai Province, China; b Tibet Yunwang Industrial Co., Ltd., Shigatse, Tibet, China; c Northwest Institute of Plateau Biology, University of the Chinese Academy of Sciences, Beijing, China; d Qinghai Academy of Animal and Veterinary Science/State Key Laboratory of Plateau Ecology and Agriculture, Qinghai University, Xining, Qinghai Province, China

**Keywords:** *Trikeraia hookeri*, chloroplast genome, Poaceae, phylogenetic analysis

## Abstract

*Trikeraia hookeri* is an alpine grass with significant ecological value. Here, the complete chloroplast genome sequence of *T. hookeri* using Illumina sequencing data was reported. The size of the whole cp genome was 137,696 bp in length, consisting of a pair of inverted repeats (IR 13,755 bp), a large single-copy region (LSC 81,613 bp), and a small single-copy region (SSC 28,568 bp). The *T. hookeri* chloroplast genome encodes 119 genes: 81 mRNA genes, 34 tRNA genes and 4 rRNA genes. The GC content of *T. hookeri* chloroplast genome was 38.8% and those in LSC, SSC, and IR regions were 36.9, 40.8, and 42.3%, respectively. The maximum-likelihood phylogenetic analysis demonstrated that *T. hookeri* was most closely related to *Stipa lipskyi* (NC028444) and *Stipa purpurrea* (NC029390). Our findings provide fundamental information for further evolutionary and phylogenetic researches of *T. hookeri*.

*Poaceae* is an important vegetation consistute part in alpine grassland community. The *Stipeae,* belonging to the subfamily *Pooideae,* family *Poaceae,* which is of special significance among the alpine steppe vegetation in Qinghai-Tibet plateau, which include approximately 400–500 species and 24 genera in all of the world, in which 67 species and 10 genera were distributed in china. *Trikeraia hookeri* is a kind of Stipeae, extremely salinity-tolerant, cold-tolerant and drought-tolerant species (Khan 2003), distributed at 4300–5400 m in Qinghai and Tibet of china (Shukla and Srivastava 2020). *T. hookeri* increased the coverage of plant community, also provided food and habitation for animals. However, no studies on the plastome of *T. hookeri* have been published. In this study, we reported the complete chloroplast genome sequence of *T. hookeri* and explored its internal relationships with the family steppe.

The seeds of *T. hookeri* was collected from Zhongba county (83°30′05″E, 30°10′38″N), Shigatse, Tibet of China. A specimen was deposited at Northwest Institute of Plateau Biology, Chinese Academy of Sciences (NWIPB, Sha Xiao, xiaosa@nwipb.cas.cn) under the voucher number 0334882. In 2020, the seed was grown in light incubator, at four-leaf stage, the leaf was extracted DNA by CTAB (Dreisigacker et al. 2012) and then sequenced using the Illumina NovaSeq platform (Illumina, San Diego, CA). The raw data were used to assemble the complete cp genome using GetOrganelle software (Jin et al. 2020), and the chloroplast genome of related species *Nassella hyalina* (GeneBank accession number NC036696) were used as the reference genome. The genome annotation was p erformed with the program Geneious R8 (Biomatters Ltd, Auckland, New Zealand). Finally, the complete chloroplast genome sequences of *T. hookeri* were submitted to GenBank (accession number: MW699773).

The whole chloroplast genome of *T. hookeri* cp genome is 137,696 bp in size, consisting of a pair of inverted repeats (IR 13,755 bp), a large single-copy region (LSC 81,613 bp), and a small single-copy region (SSC 28,568 bp). A total of 119 genes were annotated, containing 81 mRNA genes, 34 tRNA genes and 4 rRNA genes. The GC content of *T. hookeri* chloroplast genome was 38.8% and those in LSC, SSC, and IR regions were 36.9, 40.8, and 42.3%, respectively.

To confirm the phylogenetic location of *T. hookeri*, 11 genera of complete chloroplast genomes from Gramineae were obtained from GenBank, and the 12 complete chloroplast sequences were aligned using MAFFT (Katoh and Standley 2013). The maximum-likelihood analysis was performed by MEGA7 (Kumar et al. 2016) with bootstrap set to 1,000 (Figure 1). The phylogenetic analysis showed a strong sister relationship with Stipa lipskyi (NC028444) and Stipa purpurrea (NC029390).

**Figure 1.**
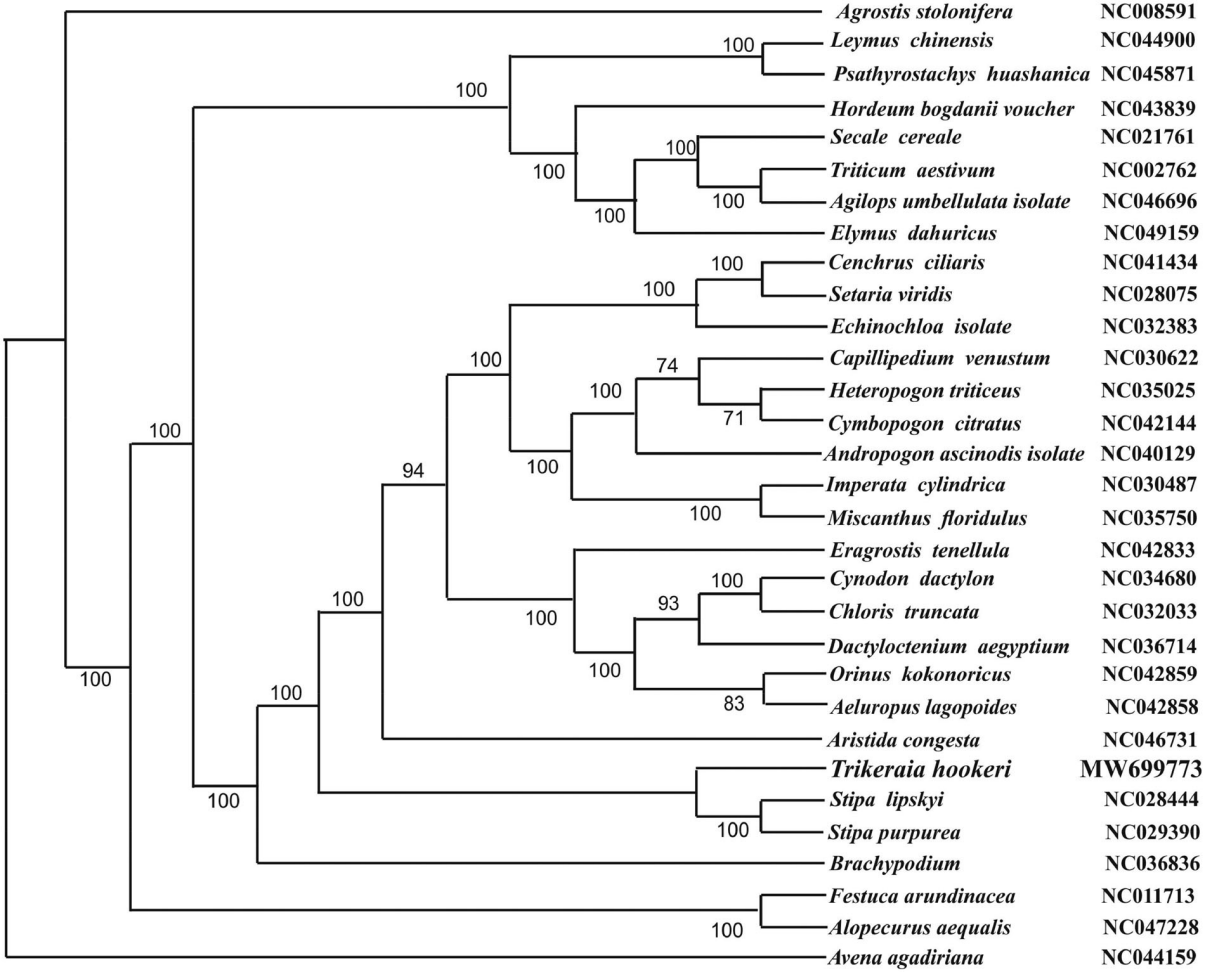
The maximum likelihood tree based on 30 complete chloroplast genome sequences. All the sequences were downloaded from NCBI GenBank. Bootstrap support values are given at the nodes.

## Data Availability

The genome sequence data that support the findings of this study are openly available in GenBank of NCBI at (https://www.ncbi.nlm.nih.gov/) under the accession no. MW699773. The associated BioProject, SRA, and Bio-Sample numbers are PRJNA671634, SAMN16539309, and SRR12888103, respectively.

## References

[CIT0001] Dreisigacker S, Sehgal D, Luna B, Reyes AE, Mollins J. 2012. CIMMYT wheat molecular genetics laboratory protocols and applications to wheat breeding. Mexico: CIMMYT.

[CIT0002] Jin JJ, Yu WB, Yang JB, Song Y, dePamphilis CW, Yi TS, Li DZ. 2020. GetOrganelle: a fast and versatile toolkit for accurate de novo assembly of organelle genomes. Genome Biol. 21(1):241. 32912315 10.1186/s13059-020-02154-5 PMC7488116

[CIT0003] Katoh K, Standley DM. 2013. MAFFT multiple sequence alignment software version 7: improvements in performance and usability. Mol Biol Evol. 30(4):772–780. 23329690 10.1093/molbev/mst010 PMC3603318

[CIT0004] Khan MA. 2003. An ecological overview of halophytes from Pakistan. In: Kratochwil A, Lieth H, editors. Cash crop halophytes: recent studies. Netherlands: Kluwer Academic Publishers; p. 175.

[CIT0005] Kumar S, Stecher G, Tamura K. 2016. MEGA7: molecular evolutionary genetics analysis version 7.0 for bigger datasets. Mol Biol Evol. 33(7):1870–1874. 27004904 10.1093/molbev/msw054 PMC8210823

[CIT0008] Shukla N, Srivastava SK. 2020. Flora of Ladakh: an annotated inventory of flowering plants. In: Ghulam HD, Anzar AK, editors. Biodiversity of the Himalaya: Jammu and Kashmir State. Springer Nature Singapore Pte Ltd. Singapore: Springer Nature; p. P.724.

